# An in silico and in vitro pipeline for the rapid screening of helicase modulators

**DOI:** 10.14806/ej.25.0.927

**Published:** 2020-02-13

**Authors:** Eleni Papakonstantinou, Flora Bacopoulou, Vasileios Megalooikonomou, Aspasia Efthimiadou, Dimitrios Vlachakis

**Affiliations:** 1 Laboratory of Genetics, Department of Biotechnology, School of Food, Biotechnology and Development, Agricultural University of Athens, Athens, Greece; 2 Lab of Molecular Endocrinology, Center of Clinical, Experimental Surgery and Translational Research, Biomedical Research Foundation of the Academy of Athens, Athens, Greece; 3 Center for Adolescent Medicine and UNESCO Chair on Adolescent Health Care, First Department of Pediatrics, Medical School, National and Kapodistrian University of Athens, Aghia Sophia Children’s Hospital, Athens, Greece; 4 Computer Engineering and Informatics Department, School of Engineering, University of Patras, Patras, Greece; 5 Hellenic Agricultural Organization-Demeter, Institute of Soil and Water Resources, Department of Soil Science of Athens, Lycovrisi, Greece; 6 Department of Informatics, Faculty of Natural and Mathematical Sciences, King’s College London, Strand, London, Uinted Kingdom

## Abstract

To evaluate the potency of potential helicase modulators, we developed an assay of helicase enzyme activity. Using a DNA or RNA biotin labelled oligonucleotide and after the addition of a recombinant helicase, the nucleic acid unwinds, causing the emission of luminescence, which is quantified with a particular antibody. In our assay, one of the DNA oligos was biotinylated, while the other was labelled with digoxygenin (DIG), both in their 5’ termini. The biotin molecule immobilises the DNA duplex on a neutravidin-coated plate and the helicase activity is measured through the unwinding of DNA, due to ATP activation. The subsequent release of DIG-labelled oligos results in a luminescence signal measured with a chemiluminescence antibody. Our goal was to provide a high throughput screening method for potential helicase inhibitors. The method described in this paper has been demonstrated to be fast, easy and reproducible and doesn’t use radiochemicals.

## Introduction

Helicase activity assays include analysis of ATPase activity, but it was shown that measuring the helicase unwinding activity is the best method for evaluating modulators of this class of enzyme (Borowski *et al.*, 2002). This type of assay depends on the ability of the enzyme to separate the release strand of DNA or RNA from the template strand. Other methods measure the deposition of radio-labelled release strands after gel electrophoresis, thin-layered chromatography or scintillation counting (Borowski *et al.*, 2001; Bartelma and Padmanabhan 2002; Alaoui-Ismaili *et al.*, 2000). These methods could be enhanced via high-throughput screening, although the radioactive materials would be a problem. Another method detects DIG-labelled release strands by ELISA (Hsu *et al.*, 1998). We propose a combination of the methods as mentioned above without radio-labelled molecules that can detect the residual release strand with a chemiluminescent antibody, giving a robust helicase assay and a stable readout, well suited to high-throughput screening.

Our goal was to provide a high throughput screening method for potential helicase inhibitors, and that is why we developed this fast, easy and reproducible assay, without using radiochemicals. The high reproducibility of the assay is obvious after the observation of only insignificant variations on a single 96-reaction plate. Helicase from the Hepatitis C Virus (HCV) was expressed and isolated through recombinant protein methods and later used in our assay.

## Materials

All solutions were prepared using ultrapure water obtained by purifying deionised water. All reagents were stored at room temperature unless indicated otherwise.

### HCV recombinant helicase preparation

1. Escherichia coli induction: Prepare 4 conical flasks with 750 mL of LB and add 34 μg/mL chloramphenicol and 25 μg/mL kanamycin to each;
2. cell lysis buffer: 20 mM sodium phosphate pH 7.5, 300 mM NaCl;
3. buffer S: 20 mM sodium phosphate pH 7.4, 500 mM NaCl;
4. exchange buffer: 25 mM Tris–HCl pH 7.5, 0.05% CHAPS (3-[(3-cholamidopropyl)-dimethylammonio]-1-propane sulphonate), 20% glycerol, 5 mM DTT (dithiothreitol).

### HCV helicase assay procedure

1. Oligonucleotide mix:
  - oligonucleotides (5’-biotin-GCTGACCCTGCTCCCAATCGTAATCTATAGTGTCACCTA-3’,
  - 5’-DIG-CGATTGGGAGCAGGGTCAGC-3’) 1:1 molar, HEPES 2mM, NaCl 0.05M, EDTA 0.1 mM, SDS 0.01% w/v;
2. neutravidin stock solution: add neutravidin in final concentration 1 mg/ml in phosphate buffered saline (1 M PBS - pH 7.0);
3. BSA solution: 0.1% w/v BSA;
4. substrate solution: mix 2.5 ng partially annealed DNA duplex and 75μL 1 M PBS containing 1M NaCl, for each well;
5. substrate wash solution: 50 mM Tris HCl pH 7.5, 50 mM NaCl;
6. helicase reaction mix: 11 nM purified full-length HCV NS3 protein, 25 mM 4-morpholinepropanesulphonic acid (MOPS) pH 7.0, 2 mM DTT, 3mM MnCl2, 100 μg/ml of BSA and 5 mM ATP. The reaction mix for the negative control lacks ATP;
7. reaction wash solution: 150 mM NaCl;
8. detection washing buffer: 0.1 M maleic acid, 0.15 M NaCl, 0.3%, Tween20, pH 7.5;
9. blocking solution: 10% BSA w/v, 0.1 M maleic acid, 0.15 M NaCl, pH 7.5;
10. antibody solution: 1:10.000 solution of the anti-Dig antibody (75 mU/mL) in Blocking solution;
11. detection buffer: 0.1 M Tris-HCl, 0.1 M NaCl, pH 9.5;
12. chemiluminescence substrate solution: CSPD – 0.25 mM.

## Methods

All procedures have to be carried out at room temperature unless specified otherwise.

### HCV recombinant helicase NS3 preparation

1. Insert the full-length HCV helicase coding region in a pET28a vector, with a N-terminal 6xHis-Tag region;
2. verify the intactness of the gene before inducing the protein production;
3. transform Escherichia coli cells (strain C41, DE3) with the helicase plasmid and inoculate the prepared LB flasks with them;
4. induce the recombinant helicase production, adding 0.5 mM IPTG to each flask and then allow the cultures to grow for 3 hours at 18°C;
5. resuspend the cell pellet from the 4 cultures in 30 mL Lysis buffer and then add lysozyme 100μg/mL and Triton X-100 0.1%;
6. incubate on ice for 30 min and then sonicate four times for 20 sec with 15 sec intervals;
7. centrifuge the suspension at 15.000 x g for 20 min;
8. adjust the clarified homogenates to 10 mM imidazole and filter them through a 0.45 μm membrane;
9. load the homogenates twice on nickel affinity columns (Ni-NTA);
10. wash each column with five times the column volume of buffer S, containing 10 mM imidazole;
11. elute the NS3 helicase with buffer S containing 300 mM imidazole;
12. immediately after the elution, exchange the buffer in the helicase-containing fractions for the Exchange buffer, via dialysis; this step is critical in order to avoid precipitation;
13. evaluate the protein concentration using the Bradford assay with BSA as standard;
14. create aliquots of the NS3 helicase and store at −80°C.

**Figure 1.**
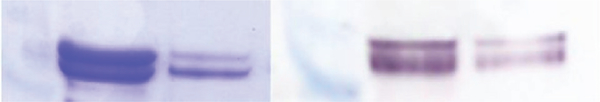
The SDS gel (left) and western blot (right, anti-His-tag antibody, penta-His conjugated) for the HCV helicase protein.

This recombinant protein preparation is estimated to be more than 85% pure by SDS gel electrophoresis and Coomassie blue staining, yielding almost 1.6 mg of HCV NS3 per 3 litres of E. coli cultures (Figure 1).

Chemiluminesence readings were taken using all different control combinations (presence/absence of helicase, DNA substrate and ATP) of the experiment to ensure the reliability of the measurements (Table 1).

**Figure 2.**
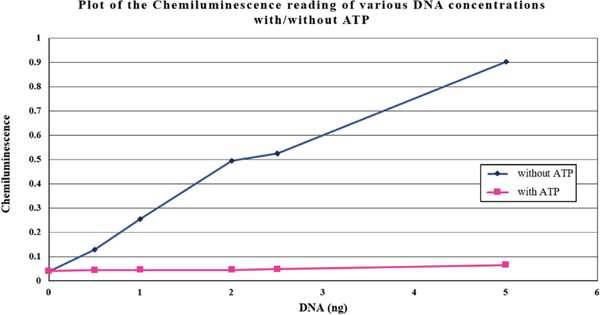
Graphical representation of Table 2 data.

We demonstrated that the NS3-mediated unwinding is proportional to the amount of DNA substrate in the well, but also to the HCV helicase concentration in the reaction. The reactions were ATP-dependent (Table 2 and Figure 2).

### Annealing reaction

1. Prepare for annealing by heating the oligonucleotide mix at 100°C for 5 min;
2. incubate at 65°C for 30 min and then at 22° for 4 h, to allow gradual annealing;
3. store the annealed NS3 helicase substrate at −20°C (See Note 1).

### Neutravidin coating of the 96-well plates

1. Coat each of the 96 wells overnight at 4°C with 100 μl/well of a 5 μg/ml neutravidin solution in 0.5 M sodium carbonate buffer pH 9.3;
2. wash the plates three times with 100μl/well of PBS and air-dry at room temperature.

### Blocking with BSA

1. Add 100 μL of the 0.1% w/v BSA solution;
2. incubate at 22°C for 2 h;
3. wash the plate three times with PBS, 200 μl/well and air-dry at room temperature;
4. store the plate at 4°C with desiccant (See Note 2).

### Substrate application in the 96-well plate

1. Pre-warm all solutions to 37°C (See Note 3);
2. mix 75 μl of the Substrate solution with 2.5 ng of the partially annealed DNA duplex for each well;
3. incubate at 22°C for 4 h;
4. wash each well twice with 200 μl PBS per well and once with Substrate Wash solution.

### Helicase Reaction

1. Add 90 μL of the Reaction mix per well;
2. incubate the reactions at 37°C for 1 hour;
3. wash the plate twice with 200 μL Reaction Washing Buffer per well and let dry for 15 minutes at room temperature.

### Activity determination – chemiluminescence preparation

1. Wash all wells for 5 min with the Detection washing buffer;
2. then fill up each well with Blocking solution for 30 min and then incubate for 30 min in 20 μl Antibody solution;
3. wash twice the plate with 100 μl of Detection buffer;
4. apply 20 μL of Detection buffer for equilibration and 1 μL of chemiluminescence substrate working solution per well and incubate for 5 min at 17°C;
5. drain the wells and incubate the plate at 37°C for 30 min to allow any remaining solution to evaporate;
6. the luminescence has a constant intensity for about 24 hours and continues for approximately 48 hours. The remaining DIG in each well is counted for 10 min against controls (one of which lacks protein and the other lacks ATP) in a luminescence plate reader (See Note 4).

## Figures and Tables

**Table 1.** Each enzymatic activity assay was performed in triplicate and the results were averaged. All reactions were allowed to proceed for 60 minutes (concentrations as described in the methods section).

	CHEMILUMINESENCE
+ DNA substrate − helicase − ATP	0.532 (± 2%)
+ DNA substrate − helicase + ATP	0.529 (± 4%)
+ DNA substrate + helicase − ATP	0.525 (± 5%)
− DNA substrate + helicase + ATP	0.040 (± 3%)

**Table 2.** Different DNA substrate concentrations (60-minute run).

DNA (ng) immobilised per well	CHEMILUMINESENCE without ATP	CHEMILUMINESENCE with ATP
0	0.039	0.040
0.5	0.128	0.044
1	0.255	0.045
2	0.495	0.045
2.5	0.525	0.048
5	0.902	0.065

## Key Points

- Testing new antiviral drugs.
- Stopping viral enzymes as therapy.
- Using supercomputers to design new drugs.
- Fast, cheap and reliable test for antiviral agents.

## Footnotes

Note 1: The oligonucleotides used in this protocol are the ones described by Alaoui-Ismaili et al. (Alaoui-Ismaili *et al.*, 2000), modified by DIG labelling of the release strand. Other sequences could work, but a 3’ single stranded region in the substrate is necessary to initiate the strand displacement (Tai *et al.*, 1996).

Note 2: As far as the blocking procedure is concerned, it is important to ensure that all potential binding sites are occupied, to prevent direct binding of the detection antibody to the well in a later step. The wells should be filled with blocking solution, fully coating the plate.

Note 3: Pre-warming the solutions allows reactions to proceed at their optimum temperatures and avoids rate changes due to temperature equilibration.

Note 4: All the reaction wells should be filled up with blocking solution, to ensure that the whole well has been blocked, preventing non-specific binding of any components of the detection system.

## References

[1] Alaoui-Ismaili MH, Gervais C, Brunette S, Gouin G, Hamel M (2000) A novel high throughput screening assay for HCV NS3 helicase activity. Antiviral Res. 46, 181–193. 10.1016/s0166-3542(00)00085-1 10867156

[2] Bartelma G & Padmanabhan R (2002) Expression, purification, and characterization of the RNA 5’-triphosphatase activity of dengue virus type 2 nonstructural protein 3. Virology 299, 122–132. 10.1006/viro.2002.1504 12167347

[3] Borowski P, Niebuhr A, Mueller O, Bretner M, Felczak K (2001) Purification and characterization of West Nile virus nucleoside triphosphatase (NTPase)/helicase: evidence for dissociation of the NTPase and helicase activities of the enzyme. J Virol 75, 3220–3229. 10.1128/JVI.75.7.3220-3229.2001 11238848 PMC114115

[4] Borowski P, Niebuhr A, Schmitz H, Hosmane RS, Bretner M (2002) NTPase/helicase of Flaviviridae: inhibitors and inhibition of the enzyme. Acta Biochim Pol 49, 597–614. http://dx.doi.org/024903597 12422230

[5] Hsu CC, Hwang LH, Huang YW, Chi WK, Chu YD (1998) An ELISA for RNA helicase activity: application as an assay of the NS3 helicase of hepatitis C virus. Biochem Biophys Res Commun 253, 594–599. 10.1006/bbrc.1998.9813 9918773

[6] Tai CL, Chi WK, Chen DS & Hwang LH (1996) The helicase activity associated with hepatitis C virus nonstructural protein 3 (NS3). J Virol 70, 8477–8484. 10.1128/JVI.70.12.8477-8484.1996 8970970 PMC190938

